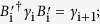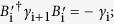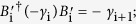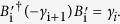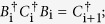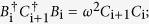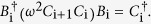# ℤ_3_ parafermionic chain emerging from Yang-Baxter equation

**DOI:** 10.1038/srep21497

**Published:** 2016-02-23

**Authors:** Li-Wei Yu, Mo-Lin Ge

**Affiliations:** 1 Theoretical Physics Division, Chern Institute of Mathematics, Nankai University, Tianjin 300071, China

## Abstract

We construct the 1D 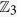 parafermionic model based on the solution of Yang-Baxter equation and express the model by three types of fermions. It is shown that the 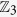 parafermionic chain possesses both triple degenerate ground states and non-trivial topological winding number. Hence, the 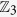 parafermionic model is a direct generalization of 1D 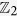 Kitaev model. Both the 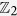 and 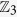 model can be obtained from Yang-Baxter equation. On the other hand, to show the algebra of parafermionic tripling intuitively, we define a new 3-body Hamiltonian 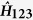 based on Yang-Baxter equation. Different from the Majorana doubling, the 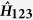 holds triple degeneracy at each of energy levels. The triple degeneracy is protected by two symmetry operators of the system, *ω*-parity *P*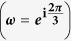 and emergent parafermionic operator Γ, which are the generalizations of parity *P*_*M*_ and emergent Majorana operator in Lee-Wilczek model, respectively. Both the 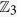 parafermionic model and 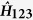 can be viewed as SU(3) models in color space. In comparison with the Majorana models for SU(2), it turns out that the SU(3) models are truly the generalization of Majorana models resultant from Yang-Baxter equation.

The double degeneracy of a pair of Majorana zero modes in condensed matter system has attracted much attentions due to its potential applications in quantum computation and quantum information^1^,^2^,^3^,^4^,^5^,^6^. It is well known that this topologically protected doubling is immune to local perturbations. Taking 1D p-wave Kitaev model^1^ as an example, the Majorana mode appears in the topological phase where the two free Majorana fermions *γ*_1_ and *γ*_2*N*_ can be excited without cost of energy at the two ends of the chain model and compose a non-local complex fermion, hence the ground state possesses double degeneracy. The two degenerate states can be differentiated by the electron number parity operator 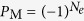, i.e., one state possesses parity −1 with odd electron occupation number, while the other possesses parity +1 with even electron occupation number. On the other hand, to give an intuitive analysis about the Majorana doubling, Lee and Wilczek^7^ proposed a 3-body Hamiltonian 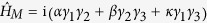, where the symmetry operators *P*_M_ and emergent Majorana operator Γ_M_ lead to the doubling at any energy level.

In our previous paper, we have shown that both the Kitaev model and the Lee-Wilczek model can be derived from the 4 × 4 matrix representation of Yang-Baxter equation(YBE)^8^. The applications of YBE^9^,^10^,^11^,^12^,^13^,^14^ in constructing many body Hamiltonian had been discussed in various papers^15^,^16^,^17^,^18^. Specifically, based on the Majorana representation of Yang-Baxter equation 
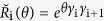
, we take the time derivative of *θ* in 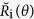 to obtain the 1D Kitaev model^8^. To self-contain, we first recall some results related to 
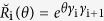
 which emerges from the 4 × 4 matrix representation of YBE, which reads


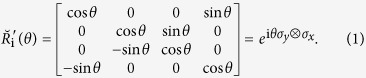


When 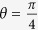, the 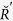-matrix turns into the braid operator *B*′^19^


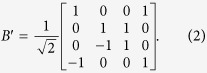


In our previous paper^8^, we have shown that the Majorana representation relates to the 4 × 4 matrix representation of YBE 
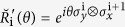
 in tensor product space through Jordan-Wigner(J-W) transformation, here *σ*_*x*_ and *σ*_*y*_ are Pauli matrices, i and i + 1 signify lattice sites. The J-W transformation transforms spin-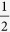 operators at lattice sites into spinless fermions through


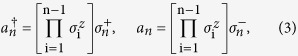


where 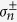 are spin ladder operators, 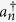 and *a*_*n*_ are spinless fermions. Define the Majorana fermion^1^


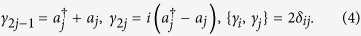


Substituting equation (3) and (4) into equation (1), we can express equation (1) as next nearest neighbor interaction of Majorana operators,


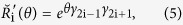


where *γ*_2i−1_ satisfy Clifford algebra {*γ*_2i−1_, *γ*_2j−1_} = 2*δ*_ij_. Based on the Clifford algebra, the 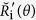 can be rewritten as


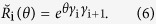


It is easy to check that the 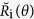 satisfies YBE. Hence the matrix representation of solution of YBE and Majorana representation of braid operators are well related.

A question is raised naturally. One spin site corresponds to 2 subcells of Majorana fermions that are related to 4 × 4 YBE in the tensor space due to the 4-d representation of Temperley-Lieb algebra. On the other hand, the 9 × 9 form of solution of YBE has been known, then could we extend the above discussion to the new type of “Majorana fermions” with 3 subcells on one spin site? The answer is yes. Instead of SU(2), the SU(3) operators should naturally be introduced. For the convenience, we call the space color space.

Since the Majorana models hold 2-fold degeneracy, for SU(3), how can we extend the Majorana double degeneracy to triple degeneracy? In other words, can we construct the extended 1D Kitaev model holding triple degenerate ground state? Indeed, similar to those in constructing Majorana models via 4 × 4 matrix solution of YBE, we can find the triple degenerate models based on the 9 × 9 matrix representation of YBE^20^.

In this paper, we make the following progress: 1) Based on the 9 × 9 matrix representation of YBE and the 3 × 3 3-cyclic representation of SU(3) generators (see Supplementary), we make the decomposition of the 9 × 9 matrix by tensor products of 3-dimensional matrices. By defining generalized SU(3) J-W transformation, we transform the SU(3) sites into non-local operators and obtain the new representation of YBE. 2) We obtain the 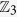 parafermionic chain with triple degenerate ground states in color space and express the chain with three types of fermions, besides that, the 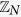 case is discussed; 3) In 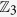 parafermionic model, the topological phase transition is signified by the triple degeneracy of ground states and the topological winding number; 4) To give an intuitive explanation of the triple degeneracy and analyse the algebraic structure in it, we construct a 3-body Hamiltonian and find its symmetry operators that lead to the tripling.

## Results

### Review of two Majorana models

To preserve the self-consistency of this paper, firstly, let us give a brief introduction to the construction of Majorana models based on YBE. The intrinsic connection between the solution 
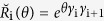
 of YBE and Kitaev model is that both of them possess 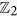 symmetry. Next we review the Kitaev model derived from YBE.

We imagine that a unitary evolution is governed by 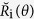. If only *θ* (tan *θ* is the velocity *u* of a particle) in unitary operator 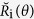 is time-dependent, we can express a state 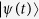 as 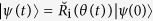. Taking the Schrödinger equation 
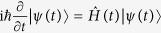
 into account, one obtains:


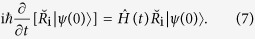


Then the Hamiltonian 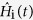 related to the unitary operator 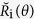 is given by:


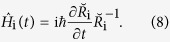


Substituting 
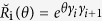
 into equation (8), we have


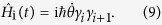


If we only consider the nearest-neighbour interactions between two Majorana fermions(MFs) and extend equation (9) to an inhomogeneous chain with 2N sites, the derived chain model is expressed as^8^:


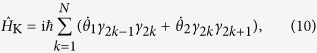


with 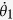 and 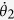 describing odd-even and even-odd pairs, respectively. This is exactly the Kitaev model derived from YBE.

The properties of 1D Kitaev model are well known:

1. In the case 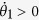, 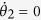, the Hamiltonian reads:


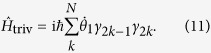


As defined in equation (4), the Majorana operators *γ*_2*k*−1_ and *γ*_2*k*_ come from the same ordinary fermion site k, 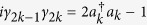 (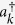 and *a*_*k*_ are spinless ordinary fermion operators). 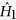 simply means the total occupancy of ordinary fermions in the chain and has U(1) symmetry, *a*_*j*_ → *e*^*iϕ*^*a*_*j*_. The ground state represents the ordinary fermion occupation number 0. This Hamiltonian corresponds to the trivial case of Kitaev’s.

2. In the case 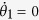, 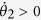, the Hamiltonian reads:


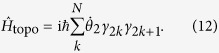


This Hamiltonian corresponds to the topological phase of 1D Kitaev model and has 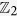 symmetry, *a*_*j*_ → −*a*_*j*_. Here the operators *γ*_1_ and *γ*_2*N*_ are absent in 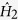. The Hamiltonian has two degenerate ground state, 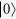 and 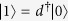, *d*^†^ = (*γ*_1_ − *iγ*_2*N*_)/2. This mode is the so-called Majorana mode in 1D Kitaev model.

On the other hand, as pointed out by Lee and Wilczek in ref. 7, the double degeneracy of Majorana models 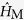 is due to two symmetry operators, the parity operator *P*_M_ and emergent Majorana operator Γ_M_. For instance, in 3-body Majorana model with the Hamiltonian


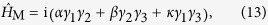


the symmetry operators and commutation relations are


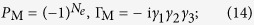



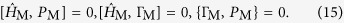


Clearly, in the basis of both 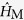 and *P*_M_ are diagonal, Γ_M_ transforms the states with *P*_M_ = ±1 into the states with 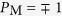. Therefore the Hamiltonian possesses Majorana doubling.

### Yang-Baxter equation and 3-cyclic SU(3) generators

Since the YBE is properly applied in constructing 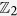 Kitaev model, we try to extend the result to 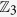-symmetric model. Fortunately, the known 9 × 9 matrix representation of the solution to YBE is a proper unitary operator for constructing the desired model with 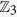 symmetry. Now we give a brief introduction to the 9 × 9 matrix representation of the solution to YBE which is associated with this paper. Firstly, let us introduce the braid matrix^20^ for 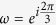:


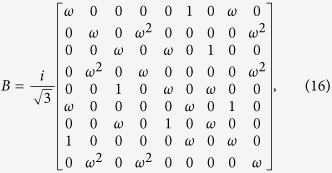


which satisfies the braid relation


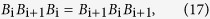


where *B*_*i*_ = *I* ⊗... *I* ⊗ *B*_*i*,*i*+1_ ⊗ *I*... (*I* is 3 × 3 identity matrix).

The solution 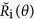 of Yang-Baxter equation can be viewed as the parametrization of braid operators,


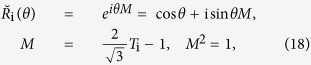


where 
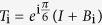
 satisfies the Temperley-Lieb algebraic (TLA) relation^21^


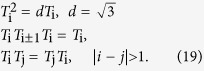


Then the YBE^22^ means that a 3-body S-matrix can be expressed in terms of three 2-body S-matrices, i.e.:


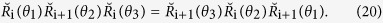


Substituting equation (18) into equation (20), we have the constraint for three parameters *θ*_1_, *θ*_2_ and *θ*_3_ :


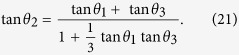


When 
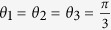
, the YBE turns into the braid relation with 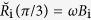. Note that due to the different *d* in TLA for 4 × 4 and 9 × 9 solutions of YBE (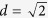 for 4 × 4), the angular relation in equation (21) for 9 × 9 is different from the angular relation for 4 × 4 in equation (1),


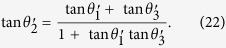


It is well known that the physical meaning of tan *θ*_I_ (or 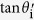) for *i* = 1, 2, 3 is the velocity of a particle. The angular relation for 4 × 4 solution means the Lorentz addition of the velocity.

By introducing the 3-cyclic SU(3) generators 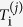 based on the principal representation of V. Kac^23^ (see Supplementary), TLA generator can be expressed as the tensor product of nearest SU(3)-lattice sites,


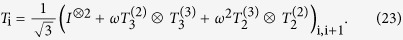


Here^24^


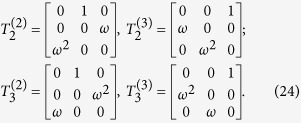


Hence at each lattice site there is one SU(3) operator which can be identified with the colors blue, red and green^25^. In the following sections, we will make use of equation (23) to generate the topological non-trivial models and triple degeneracy.

### Ladder operators of SU(3) spin and extended Jordan-Wigner transformation

In this section, we present the ladder operators of SU(3) spin and introduce the extended Jordan-Wigner transformation for SU(3) spin sites.

For spin-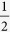 at lattice sites expressed by SU(2) Pauli matrices, the ladder operators are


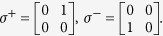


Similarly, we introduce the cyclic ladder operators of SU(3) spin


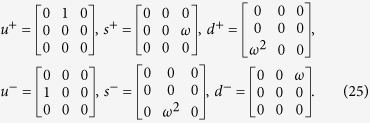


The above operators act on the color space to transform the three colors into each other obeying the algebraic relations


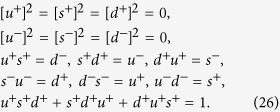


Unlike SU(2) spin, the SU(3) spin has 3 independent ladder operators *u*^+^, *s*^+^ and *d*^+^ to make the transition between three different colors. *u*, *s* and *d* can be expressed by *u*^+^, *s*^+^ and *d*^+^.

To introduce the extended Jordan-Wigner transformation for SU(3), let us review the original SU(2) J-W transformation firstly. J-W transformation transforms sited spin-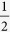 operators onto spinless fermions


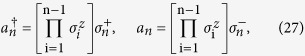


where 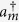 and *a*_*m*_ satisfy the fermionic commutation relations


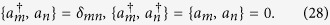


From the above transformation, it turns out that the anti commuting relations of fermions result from 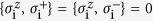.

Similarly, the extended J-W transformation for SU(3) can be defined as^26^


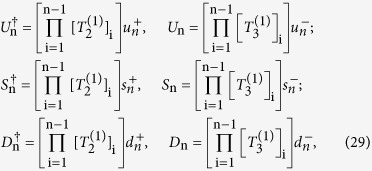


where^24^


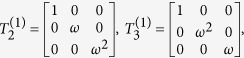


in which it follows


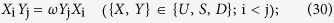



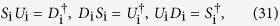


that can be checked straightforwardly. Different from the anti commuting of spinless fermions, in the exchange between the above operators there appear extra *ω* (or *ω*^2^) phase factor. The physical meaning is obvious, when making exchange between two particles on *i*-th and *j*-th sites (*i* < *j*), the system gains an extra *ω* phase factor. Exchanging the two particles again, the system returns to the initial state.

By introducing the linear combination of sited SU(3) operators


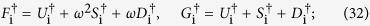



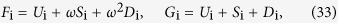



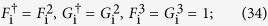



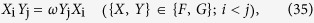


the TLA generator *T*_i_ in equation (23) can be rewritten as


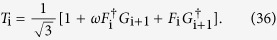


Here 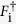 and 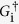 are non-local operators. Indeed, they are the generalizations of Clifford algebra which corresponds to Majorana fermions. Redefining


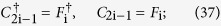



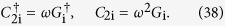


Thus the extended generator of TLA shown in equation (36) can be written in terms of the form


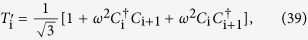


with


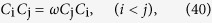



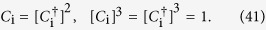


For convenience we call the commutation relation shown in equation (40) *ω*-commutation relation. This commutation relation can be regarded as the generalization of Majorana fermions’ anti-commuting, which is also proposed in^27^. Note that 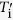 does not equal to *T*_i_, but it also satisfies 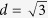 TLA in equation (19) and can be substituted into equation (18). In 1D Kitaev model, two real Majorana operators corresponds to one complex fermion site as well as one SU(2) spin site. Similarly, the two *ω*-commuting operators *C*_2i−1_ and *C*_2i_ correspond to the *i*-th SU(3) spin. Obviously, equation (40) looks *q*-commutation relation for *q*^3^ = 1 in quantum algebra^28^.

### Generating 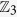 parafermionic model from YBE

From equation (18) and equation (39), we obtain the unitary solution 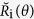 of YBE in the form


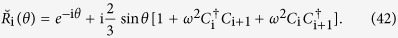


Now let us construct the 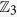 parafermionic chain based on equation (42). Substituting equation (42) into equation (8), we get


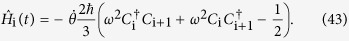


Similarly, we consider the nearest-neighbour interactions of *C*_i_’s and extend equation (43) to an 2N-chain and ignore the constant term, the derived chain model can be expressed as:


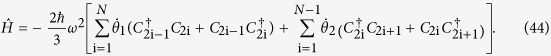


Here we emphasize that the chain possesses open boundary condition. This model is the 1D 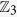 parafermionic model^26^, which originates from the three-state Potts model^29^,^30^,^31^. Instead of the 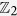 parity symmetry of Kitaev model, the model in equation (44) possesses 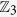 symmetry. The symmetry operator is


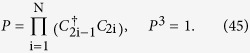


Hence *P* is a 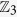 symmetry of the model and the eigenvalues of *P* is 1, *ω* and *ω*^2^. Next we analyse the obtained model in two cases.

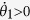
, 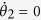.In this case the Hamiltonian becomes:
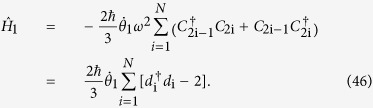
Here we note that *C*_2i−1_ and *C*_2i_ correspond to i-th SU(3) spin, 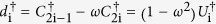,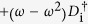 and the vacuum state 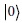 is defined as 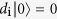. The Hamiltonian is diagonalised and the ground state is unique. This is a trivial case.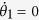, 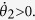
In this case the Hamiltonian is:
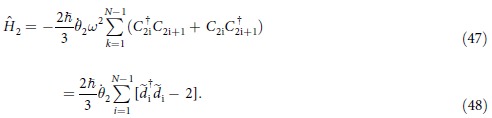
Here the quasiparticle at lattice can be defined as 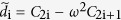. The ground states satisfy the condition 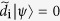 for *i* = 1,..., *N* − 1. Under the open boundary condition, it shows that the absent operators *C*_1_, 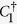, *C*_2N_ and 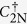 in 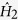 remain unpaired and are the symmetry operators of the Hamiltonian 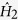. Together with the *ω*-parity operator *P*, *C*_1_, 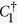, *C*_2N_ and 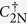 lead to the triple degeneracy of ground states which can be categorized according to *P*. The Hamiltonian has three degenerate ground states: 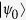, 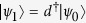 and 
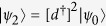
, where 
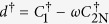
. The three ground states 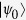, 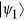 and 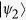 possess the parity 1, *ω* and *ω*^2^, respectively.

From the above discussion we see that the 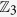 parafermionic chain is natural generalization of the 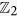 Kitaev model. Next let us construct the topological invariant for the parafermionic chain and discuss its phase transition. In terms of Fourier transformation,


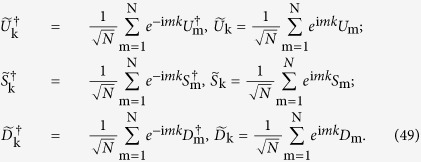


The parafermionic operators in momentum space can be written in the following form


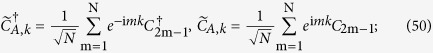



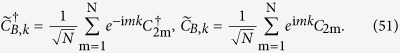


Then the equation (44) can be expressed in momentum space as


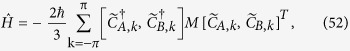


where *M* is 2 × 2 matrix,


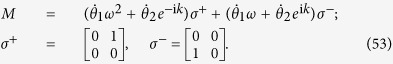


Here we note that 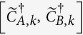 is analogous to the Majorana operators in momentum space in 1D Kitaev model. If one define [*σ*^*x*^, *σ*^*y*^, *σ*^*z*^] as a SU(2) space, *M* can be regarded as a vector in XY-plane of SU(2) space with the basis *σ*^*x*^ and *σ*^*y*^ (*k* ∈ [−*π*, *π*]), namely,


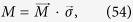



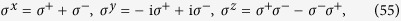



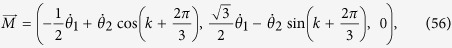



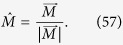


Now the topological invariant for vector 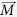 can be defined^32^,


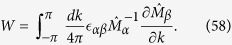


Indeed, the topological invariant *W* means the winding number of the vector 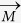 winding around the original point in the first Brillouin zone. In ref. 26, the author emphasized that the energy spectrum of parafermionic model can not be obtained simply by Fourier transformation due to the relation in equation (40). Here we do not expect to obtain the energy spectrum, but in the momentum basis of  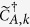 and  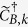, the topological winding number of 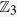 parafermionic chain shows the analogous characteristic as the 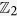 Kitaev chain. When 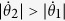, the winding number *W* = −1 corresponds to the topological non-trivial phase. When 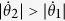, the winding number *W* = 0 corresponds to the topological trivial phase. In this sense, 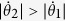 is the phase transition point. By calculating the eigenvalues of *M*, we can find that the “bulk gap” closes at 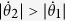 where the “bulk gap” closes, the phase transition occurs. Thus we see from the above definition that the critical point of the phase transition 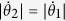 coincides with the 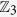 conformal field theory(CFT)^33^,^34^,^35^. Obviously, the above properties in our derived 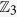 parafermionic chain are very similar to 1D Kitaev model. However, there are still some differences between 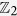 and 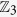 models. The critical point of 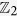 Kitaev model can be described by Ising CFT. When Kitaev model is in topological phase, it appears Majorana zero mode with quantum dimension 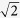. While the critical point of 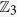 parafermion model is also described by 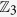 parafermion CFT, but the non-abelian primary fields are not 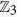 parafermion field. There are totally six different quasiparticles in 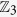 parafermion model, three of which possess abelian fields, the vacuum 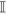, parafermion field *ψ* and *ψ*^†^. Besides, there exist three types of non-abelian fields, *σ*, *σ*^†^, 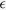, where *σ* is the spin field and 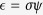 is the Fibonacci anyon with quantum qimension 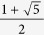. For example, the 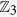 Read-Rezayi quantum Hall phase supports Fibonacci anyons, which are applicable to universal quantum computation^35^,^36^,^37^.

Now let us discuss the generalization of the cyclic chain model from 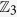 to 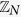. To start with, let us introduce the irreducible cyclic representation of SU(N) generators. Under the N-dimensional orthonormal basis 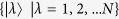, akin to SU(3), the ket-bra representation of *N*^2^ − 1 SU(N) generators are


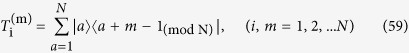



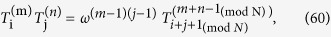


with *ω* root of unity *ω*^*N*^ = 1 and 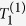 identity operator. Then the representation of Temperley-Lieb algebra *T*_*i*_ on (i, i + 1)-th sites under the cyclic *SU* (*N*) operators is expressed as


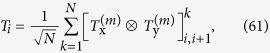


with *x*, *y*, *m*, *n* arbitrary certain integer given from 2 to N. Based on the algebraic relation in equation (60), it is easy to check that *T*_*i*_ in equation (61) satisfies TLA with quantum dimension 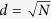,


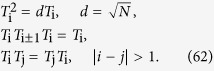


From the view point of rational Yang-Baxterization of Temperley-Lieb algebra, when *d* ≤ 2, i.e. *N* ≤ 4, the parameter in the solution of YBE is real and the corresponding 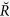-matrix is unitary and can be viewed as unitary evolution operator of a quantum system. That is, we can obtain 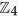 parafermion chain from YBE in an similar way as 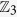. While *d* > 2, the parameter in the solution of YBE is imaginary(see Supplementary), hence the 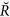 is not unitary and cannot be viewed as an ideal evolution operator. One can still construct 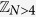 parafermion chain, but the chain does not come from the rational Yang-Baxterization.

### Fermionic representation of 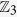 parafermionic model

In this section we express the 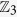 parafermionic chain in terms of fermions. For the 3 × 3 matrices in equation (25), we choose three orthonormal basis, 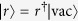, 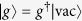 and 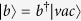. Here 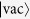 represents the vacuum state, *r*^†^, *g*^†^ and *b*^†^ are three types of fermions and satisfy the fermionic commutation conditions


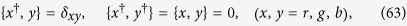


with the constraint of the occupation number of the fermions on each site


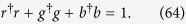


Then equation (64) means the only one occupied fermion on the site for either *r*^†^ or *g*^†^ or *b*^†^. Considering equation (25) the SU(3) ladder operators can be written as


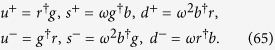


In the basis of 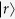, 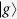 and  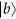, we have the relation


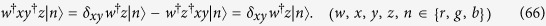


It can be proved that the operators in equation (65) satisfy the same relations as for those in equation (26) (see Supplementary). In other words, the fermionic representation of the SU(3) operators also satisfy the matrix multiplication. Similarly, the nonlocal operators *U*^†^, *S*^†^ and *D*^†^ can be expressed as


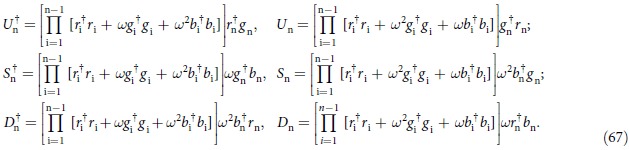


Here we do not need to require whether the commutation relation between two operators on different sites is fermionic or bosonic, since the non local operators *U*, *S* and *D* are always even power of the fermionic operators.

Making use of the fermionic representation of *U*^†^, *S*^†^ and *D*^†^ we find that 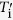 given by equation (39) also satisfies T-L algebraic relation. Then the 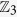 parafermionic chain is rewritten as


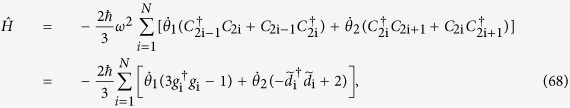


where 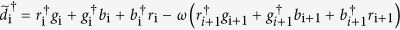. For the topological non-trivial case 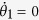, 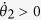, 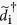 in equation (68) shows the rotation symmetry of *r*^†^, *g*^†^ and *b*^†^ for each sited SU(3) spin. For the topological trivial case 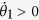, 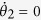, equation (68) shows that the ground state corresponds to the full occupation of fermion 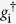 on each site. Unlike the topological non-trivial case, there is no cyclic permutation symmetry of *r*^†^, *g*^†^ and *b*^†^ on each SU(3) spin site. There are totally three types of parafermions on the i-th SU(3) spin site, 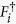, 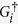 and *ωF*_*i*_*G*_*i*_ (see equation (32)), but we only choose two of the three types of parafermions to represent Temperley-Lieb algebra in equation (36). Hence there are three different ways to choose the representation of TLA as well as the 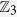 parafermionic model. Each way corresponds to one type of ground state occupation (*r*^†^ or *g*^†^ or *b*^†^).

### Algebra of triple degenerate model

In previous sections, we have discussed the 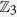 parafermionic model and the physical consequences. There appears the triple degeneracy in ground state and the emergence of tripling corresponds to the topological phase of the Hamiltonian. It can be regarded as the extension of the algebra of Majorana doubling pointed out in ref. 7. In this section, we shall show the algebra of triple degeneracy at each energy level due to the 3-cyclic and give its intuitive explanation.

Firstly let us construct 3-body Hamiltonian based on the 3-body S-matrix constrained by YBE. It is well known that the physical meaning of 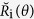 is 2-body S-matrix. YBE means that a 3-body S-matrix can be decomposed into three 2-body S-matrices in the following way


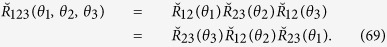


Here we note that due to the constraint of equation (21), only two of the three parameters *θ*_1_, *θ*_2_ and *θ*_3_ are free. Suppose *θ*_1_ and *θ*_2_ are time dependent, then the 3-body Hamiltonian can be obtained from equation (8) (see Supplementary)


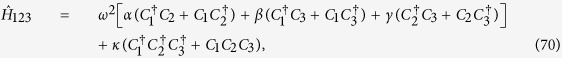


where *α*, *β*, *γ* and *κ* are real parameters depending on *θ*_1_ and *θ*_2_. By making inverse Jordan-Wigner transformation for SU(3) to transform *C*_i_’s back into SU(3) spin sites, one can show that there are only two independent symmetry operators (see Supplementary)


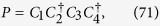



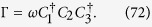


Then the complete set of the algebra for the Hamiltonian is


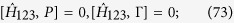



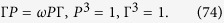


Here *P* represents the *ω*-parity operator. Now we turn to the analysis of the degeneracy of the Hamiltonian. From equation (74), Γ transforms the common eigenstates 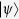 of 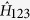 and *P* to the following form:


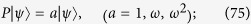



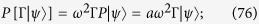



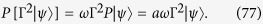


Because Γ commutes with the Hamiltonian 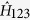, the above three states have the same energy with different *ω*-parity. As a consequence the Hamiltonian possesses triple degeneracy on all energy levels. In this sense, we conclude that 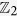 parity leads to Majorana doubling, whereas the 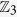 *ω*-parity leads to the tripling.

## Discussion

Before ending the paper, we would like to make some comments and discussions.

1. In our SU(3) models, only the three operators *U*^†^, *S*^†^ and *D*^†^ are basic operators that are extention of the spinless fermion *a*^†^ and *a* in SU(2) Majorana models. *U*, *S* and *D* can be expressed by *U*^†^, *S*^†^ and *D*^†^ (see equation (31)).
2. Our results can be regarded as the direct generalization of 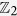 Majorana models. The comparison between 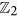 Majorana fermion and 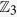 parafermion is shown in the Table 1. In 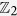 case, the eigenvalues of the symmetry operators are ±1 whereas the eigenvalues turn into 1, *ω* and *ω*^2^ for 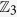 symmetry operators. We see from the Table 1 that the exchange of 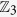 quasiparticles *C*_i_ emerges an extra *ω* or *ω*^2^ phase factor instead of −1.
3. The topological case of 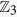 parafermionic model has been obtained, which corresponds to the triple degenerate ground states and the non-trivial topological winding number. Although the 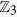 parafermionic model consists of SU(3) spin, the matrix *M* of the Hamiltonian in momentum space still forms SU(2)(see equation (53)). Hence we can follow the original Kitaev model to define the topological winding number for the 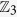 parafermionic model. The 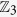 parafermionic model is formed by three types of fermions *r*^†^, *g*^†^ and *b*^†^. For topological trivial case, the ground state corresponds to the full occupation of *g*-fermion. For topological non-trivial case, the ground state shows the cyclic rotational symmetry of *r*, *g* and *b*-fermions. This may be helpful to realize the 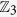 parafermionic model experimentally.
4. To give an intuitive explanation about the triple degeneracy, we construct the 3-body Hamiltonian 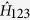 based on YBE. This model is the generalization of the 3-MF model pointed by Lee and Wilczek. Two independent symmetry operators Γ and *P* of the Hamiltonian have been found and we show that all the energy level of 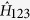 possesses triple degeneracy. In the process of constructing triple degeneracy, the 3-cyclic property of the 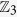 symmetry operators plays the crucial role.
5. Both of the two derived models are based on the 9 × 9 braid matrix *B*_*i*_ as well as the solution 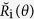 of YBE. Regarding 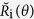 as the time dependent unitary evolution of 2-body interaction, one can construct the local 2-body interacting Hamiltonian. The 2-body Hamiltonian is then extended to the desired 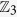 parafermionic chain. To obtain the 3-body Hamiltonian, we suppose that 3-body S-matrix can be decomposed into three 2-body S-matrices via YBE that is acceptable in low-energy physics. The advantage is in that the 3-body Hamiltonian inherits the *ω*-parity symmetry from 2-body Hamiltonian. In other words, the *ω*-parity symmetry of 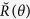 preserves the symmetry properties of 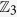 parafermionic model and 3-body Hamiltonian. This is the important role of YBE plays in obtaining the desired models.

To summarize, we extend the 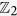 Kitaev model to 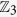 parafermionic model. Due to the 3-fold 3-cyclic, 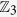 model possesses triple degeneracy. But both 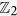 and 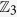 models have the similar topological phase transition scheme obtained from YBE. In this sense, YBE is a powerful tool for generating new models. How to make full use of YBE to generate more meaningful models is still a challenge problem.

## Additional Information

**How to cite this article**: Yu, L.-W. and Ge, M.-L. 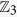 Parafermionic Chain Emerging From Yang-Baxter Equation. *Sci. Rep.* **6**, 21497; doi: 10.1038/srep21497 (2016).

## Supplementary Material

Supplementary Information

## Figures and Tables

**Table 1:** Comparison between SU(2) and SU(3) pictures.

Type of spin sites	SU(2)	SU(3)
Ladder operators	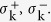	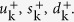
After J-W transform.	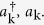	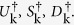
Quasiparticle	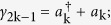 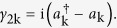 (Majorana fermions)	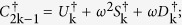 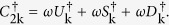
Commutation relation	*γ*_i_*γ*_j_ = −*γ*_j_*γ*_i_; 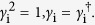	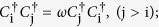 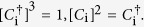
Braid operator	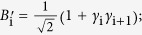 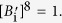	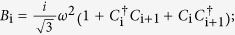 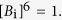 .
Cyclic operation