# Supplementary material for: ℤ3 parafermionic chain emerging from Yang-Baxter equation
Source: Sci Rep. 2016 Feb 23;6:21497. doi: 10.1038/srep21497 (PMC4794089; doi:10.1038/srep21497)
Supplement: Supplementary Information [file srep21497-s1.pdf]

Supplementary for " $\mathbb{Z}_3$ parafermionic chain emerging from Yang-Baxter equation"

Li-Wei Yu and Mo-Lin Ge
Theoretical Physics Division, Chern Institute of Mathematics, Nankai University, Tianjin 300071, China

I. ω-CYCLIC SU(3) GENERATOR IN YANG-BAXTER EQUATION

The ω-cyclic representation of SU(3) generators are (except $T_1^{(1)}$)[1]:

$$T_1^{(1)} = \begin{bmatrix} 1 & 0 & 0 \\ 0 & 1 & 0 \\ 0 & 0 & 1 \end{bmatrix}, T_1^{(2)} = \begin{bmatrix} 0 & 1 & 0 \\ 0 & 0 & 1 \\ 1 & 0 & 0 \end{bmatrix}, T_1^{(3)} = \begin{bmatrix} 0 & 0 & 1 \\ 1 & 0 & 0 \\ 0 & 1 & 0 \end{bmatrix};$$
$$T_2^{(1)} = \begin{bmatrix} 1 & 0 & 0 \\ 0 & \omega & 0 \\ 0 & 0 & \omega^2 \end{bmatrix}, T_2^{(2)} = \begin{bmatrix} 0 & 1 & 0 \\ 0 & 0 & \omega \\ \omega^2 & 0 & 0 \end{bmatrix}, T_2^{(3)} = \begin{bmatrix} 0 & 0 & 1 \\ \omega & 0 & 0 \\ 0 & \omega^2 & 0 \end{bmatrix};$$
$$T_3^{(1)} = \begin{bmatrix} 1 & 0 & 0 \\ 0 & \omega^2 & 0 \\ 0 & 0 & \omega \end{bmatrix}, T_3^{(2)} = \begin{bmatrix} 0 & 1 & 0 \\ 0 & 0 & \omega^2 \\ \omega & 0 & 0 \end{bmatrix}, T_3^{(3)} = \begin{bmatrix} 0 & 0 & 1 \\ \omega^2 & 0 & 0 \\ 0 & \omega & 0 \end{bmatrix}.$$

and they satisfy the following algebraic relations

$$\begin{aligned} &T_{\rm i}^{(\rm m)} T_{\rm j}^{(\rm n)} = \omega^{(\rm m\text{-}1)(\rm j\text{-}1)} T_a^{(b)} \quad (\omega = e^{\rm i \frac{2\pi}{3}}), \\ &a = \text{i+j-1} (\textbf{mod}\, 3),\ b = \text{m+n-1} (\textbf{mod}\, 3). \end{aligned} \tag{1}$$

The braid operator can be parametrized to yield the solution of YBE by means of Yang-Baxterization[2]. Now let us recall the standard method of Yang-Baxterization. The YBE reads,

$$\breve{R}_{\rm i}(\mu)\breve{R}_{\rm i+1}(f(\mu,\nu))\breve{R}_{\rm i}(\nu) = \breve{R}_{\rm i+1}(\nu)\breve{R}_{\rm i}(f(\mu,\nu))\breve{R}_{\rm i+1}(\mu), \tag{2}$$

and the solution is

$$\breve{R}_{\rm i}(\mu) = \rho(\mu)[1+G(\mu)T_{\rm i}], \tag{3}$$
$$G(\mu) = \frac{\mu}{a_0 - d\mu/2}, \tag{4}$$
$$f(\mu,\nu) = \frac{\mu+\nu}{1+\beta^2\mu\nu},\ \beta^2 = \frac{d^2-4}{(2a_0)^2}. \tag{5}$$

Here $T_{\rm i}$ is Temperley-Lieb algebra(TLA) generator, d represents the loop value of TLA and a_0 is a free parameter. If we express $T_{\rm i}$ in terms of the known braid operator $B_{\rm i}$, then the solution of YBE can be obtained. In this paper, the T-L generator associated with braid operators $B_{\rm i}$ can be expressed as follows,

$$\begin{aligned} T_{\rm i} &= e^{\rm i\frac{\pi}{6}}(I^{\otimes 2}+B_{\rm i}) \\ &= \frac{1}{\sqrt{3}}(I^{\otimes 2}+\omega T_3^{(2)}\otimes T_3^{(3)}+\omega^2 T_2^{(3)}\otimes T_2^{(2)})_{\rm i,i+1}. \end{aligned} \tag{6}$$

$T_{\rm i}$ satisfy T-L algebra with the loop value $d=\sqrt{3}$,

$$\begin{aligned} &T_{\rm i}^2 = dT_{\rm i}, d=\sqrt{3} \\ &T_{\rm i}T_{\rm i\pm 1}T_{\rm i} = T_{\rm i}, \\ &T_{\rm i}T_{\rm j} = T_{\rm j}T_{\rm i}, \quad |i-j|>1. \end{aligned} \tag{7}$$

Based on the algebraic relation in equation (1), we can verify these relations by direct calculation.

After the replacement

$$\tan\theta_1 = \beta\frac{{\rm i}d}{\sqrt{d^2-4}}u, \tag{8}$$
$$\tan\theta_2 = \beta\frac{{\rm i}d}{\sqrt{d^2-4}}f(u,v), \tag{9}$$
$$\tan\theta_3 = \beta\frac{{\rm i}d}{\sqrt{d^2-4}}v,\ (d=\sqrt{3}) \tag{10}$$

the solution $\breve{R}_{\rm i}(u)$ is rewritten as

$$\breve{R}_{\rm i}(\theta) = \cos\theta I + {\rm i}\sin\theta(\frac{2}{\sqrt{3}}T_{\rm i} - I), \tag{11}$$

with the angular relation

$$\tan\theta_2 = \frac{\tan\theta_1 + \tan\theta_3}{1 + \frac{1}{3}\tan\theta_1\tan\theta_3}. \tag{12}$$

When $\theta_1 = \theta_2 = \theta_3 = \frac{\pi}{3}$, the YBE turns back into the braid relation with $\breve{R}_{\rm i}(\pi/3) = \omega B_{\rm i}$.

In comparison with $d = \sqrt{2}$ for $\breve{R}(\mu)$ related to the Bell basis [3], $\mu = \tan\theta$, and

$$\mu_2 = \frac{\mu_1 + \mu_3}{1 + \mu_1\mu_3}.$$

II. PROOF OF OPERATOR MULTIPLICATION FOR $u^{\pm}, s^{\pm}, d^{\pm}$ IN THE FERMIONIC REPRESENTATION

In the orthonormal basis of $|r\rangle = r^\dagger|{\rm vac}\rangle$, $|g\rangle = g^\dagger|{\rm vac}\rangle$ and $|b\rangle = b^\dagger|{\rm vac}\rangle$, with the fermionic condition on each site

$$\{x^\dagger, y\} = \delta_{xy}, \quad \{x^\dagger, y^\dagger\} = \{x, y\} = 0, \qquad (x, y = r, g, b), \tag{13}$$

the operators $u^{\pm}, s^{\pm}, d^{\pm}$ are expressed as

$$\begin{aligned} u^+ &= r^\dagger g,\ s^+ = \omega g^\dagger b,\ d^+ = \omega^2 b^\dagger r, \\ u^- &= g^\dagger r,\ s^- = \omega^2 b^\dagger g,\ d^- = \omega r^\dagger b. \end{aligned} \tag{14}$$

Due to the constraint that the total occupation number for the fermions r, g, b is 1 on each site,

$$r^\dagger r + g^\dagger g + b^\dagger b = 1. \tag{15}$$

In the basis of $|r\rangle$, $|g\rangle$ and $|b\rangle$, we have the relation

$$w^\dagger x y^\dagger z|n\rangle = \delta_{xy} w^\dagger z|n\rangle - w^\dagger z^\dagger x y|n\rangle = \delta_{xy} w^\dagger z|n\rangle. \quad (w, x, y, z, n \in \{r, g, b\}) \tag{16}$$

i.e. acting two adjoint annihilation operators on the single fermionic occupation basis. Then multiplication of the operators $u^{\pm}, s^{\pm}, d^{\pm}$ are easily checked in the basis of $|r\rangle$, $|g\rangle$ and $|b\rangle$,

$$\begin{aligned} [u^+]^2 &= r^\dagger g r^\dagger g = -r^\dagger r^\dagger g g = 0; \\ [u^-]^2 &= g^\dagger r g^\dagger r = -g^\dagger g^\dagger r r = 0; \\ [s^+]^2 &= \omega^2 g^\dagger b g^\dagger b = -\omega^2 g^\dagger g^\dagger b b = 0; \\ [s^-]^2 &= \omega b^\dagger g b^\dagger g = -\omega b^\dagger b^\dagger g g = 0; \\ [d^+]^2 &= \omega b^\dagger r b^\dagger r = -\omega b^\dagger b^\dagger r r = 0; \\ [d^-]^2 &= \omega^2 r^\dagger b r^\dagger b = -\omega^2 r^\dagger r^\dagger b b = 0; \\ u^+ s^+ &= [r^\dagger g][\omega g^\dagger b] = \omega r^\dagger(1 - g^\dagger g)b = \omega r^\dagger b = d^-; \\ s^+ d^+ &= [\omega g^\dagger b][\omega^2 b^\dagger r] = g^\dagger(1 - b^\dagger b)r = g^\dagger r = u^-; \\ d^+ u^+ &= [\omega^2 b^\dagger r][r^\dagger g] = \omega^2 b^\dagger(1 - r^\dagger r)g = \omega^2 b^\dagger g = s^-; \\ s^- u^- &= [\omega^2 b^\dagger g][g^\dagger r] = \omega^2 b^\dagger(1 - g^\dagger g)r = \omega^2 b^\dagger r = d^+; \\ d^- s^- &= [\omega r^\dagger b][\omega^2 b^\dagger g] = r^\dagger(1 - b^\dagger b)g = r^\dagger g = u^+; \\ u^- d^- &= [g^\dagger r][\omega r^\dagger b] = \omega g^\dagger(1 - r^\dagger r)b = \omega g^\dagger b = s^+. \end{aligned}$$

Hence Temperley-Lieb algebraic relation represented by fermions can also be checked.

III. DERIVATION OF 3-BODY HAMILTONIAN

The 3-body S-matrix constrained by YBE is

$$\begin{aligned}\breve{R}_{123}(\theta_1,\theta_2,\theta_3) &= \breve{R}_{12}(\theta_1)\breve{R}_{23}(\theta_2)\breve{R}_{12}(\theta_3)\\ &= \breve{R}_{23}(\theta_3)\breve{R}_{12}(\theta_2)\breve{R}_{23}(\theta_1).\end{aligned} \tag{17}$$

Regarding $\breve{R}_{123}$ as the unitary evolution of system, one can construct the 3-body Hamiltonian

$$\hat{H}_{123}(t) = \mathrm{i}\hbar\frac{\partial \breve{R}_{123}}{\partial t}\breve{R}_{123}^{-1}. \tag{18}$$

Here we note that due to the constraint of equation (12), there are two of the three parameters θ_1, θ_2 and θ_3 are free Supposing that θ_1 and θ_2 is time dependent, we obtain

$$\begin{aligned}\hat{H}_{123} =& \mathrm{i}\hbar\dot{\theta}_1\frac{\partial \breve{R}_{12}(\theta_1)}{\partial\theta_1}\breve{R}_{12}^{-1}(\theta_1)+\mathrm{i}\hbar\dot{\theta}_2\breve{R}_{12}(\theta_1)\left[\frac{\partial \breve{R}_{23}(\theta_2)}{\partial\theta_2}\breve{R}_{23}^{-1}(\theta_2)\right]\breve{R}_{12}^{-1}(\theta_1)\\ =& -\hbar\dot{\theta}_1(\tfrac{3}{\sqrt{2}}T_1-1)-\hbar\dot{\theta}_2\breve{R}_{12}(\theta_1)\left[\tfrac{3}{\sqrt{2}}T_2-1\right]\breve{R}_{12}^{-1}(\theta_1)\\ =& -\tfrac{8}{9}\hbar\dot{\theta}_2\sin\theta_1\sin(\theta_1+\pi/3)\omega^2(C_1^\dagger C_3+C_1C_3^\dagger)+\hbar\dot{\theta}_2(\tfrac{8}{9}\sin^2\theta_1-\tfrac{2}{3})\omega^2(C_2^\dagger C_3+C_2C_3^\dagger)\\ &-\tfrac{8}{9}\hbar\dot{\theta}_2\sin\theta_1\sin(\theta_1-\pi/3)(C_1^\dagger C_2^\dagger C_3^\dagger+C_1C_2C_3)-\tfrac{2}{3}\hbar\dot{\theta}_1\omega^2(C_1^\dagger C_2+C_1C_2^\dagger)+\hbar\tfrac{1}{3}(\dot{\theta}_1+\dot{\theta}_2).\end{aligned} \tag{19}$$

Ignoring the constant term, we have

$$\begin{aligned}\alpha &= -\tfrac{2}{3}\hbar\dot{\theta}_1,\\ \beta &= -\tfrac{8}{9}\hbar\dot{\theta}_2\sin\theta_1\sin(\theta_1+\pi/3),\\ \gamma &= \hbar\dot{\theta}_2(\tfrac{8}{9}\sin^2\theta_1-\tfrac{2}{3}),\\ \kappa &= -\tfrac{8}{9}\hbar\dot{\theta}_2\sin\theta_1\sin(\theta_1-\pi/3),\end{aligned}$$

for

$$\begin{aligned}\hat{H}_{123} =& \omega^2[\alpha(C_1^\dagger C_2+C_1C_2^\dagger)+\beta(C_1^\dagger C_3+C_1C_3^\dagger)\\ &+\gamma(C_2^\dagger C_3+C_2C_3^\dagger)]+\kappa(C_1^\dagger C_2^\dagger C_3^\dagger+C_1C_2C_3).\end{aligned} \tag{20}$$

IV. SYMMETRY OPERATORS OF $\hat{H}_{123}$

In this section, we show that there are only two independent symmetry operators of 3-body Hamiltonian $\hat{H}_{123}$. Let us first transform equation (19) into matrix tensor product form under SU(3) Jordan-Wigner transformation

$$\begin{aligned}U_{\mathrm{n}}^\dagger &= \left[\prod_{\mathrm{i}=1}^{\mathrm{n}-1}[T_2^{(1)}]_{\mathrm{i}}\right]u_n^+, \quad U_{\mathrm{n}} = \left[\prod_{\mathrm{i}=1}^{\mathrm{n}-1}[T_3^{(1)}]_{\mathrm{i}}\right]u_n^-;\\ S_{\mathrm{n}}^\dagger &= \left[\prod_{\mathrm{i}=1}^{\mathrm{n}-1}[T_2^{(1)}]_{\mathrm{i}}\right]s_n^+, \quad S_{\mathrm{n}} = \left[\prod_{\mathrm{i}=1}^{\mathrm{n}-1}[T_3^{(1)}]_{\mathrm{i}}\right]s_n^-;\\ D_{\mathrm{n}}^\dagger &= \left[\prod_{\mathrm{i}=1}^{\mathrm{n}-1}[T_2^{(1)}]_{\mathrm{i}}\right]d_n^+, \quad D_{\mathrm{n}} = \left[\prod_{\mathrm{i}=1}^{\mathrm{n}-1}[T_3^{(1)}]_{\mathrm{i}}\right]d_n^-.\end{aligned} \tag{21}$$

We have

$$\begin{aligned}C_1^\dagger &= T_1^{(2)}\otimes T_1^{(1)}, \quad C_1 = T_1^{(3)}\otimes T_1^{(1)};\\ C_2^\dagger &= \omega T_2^{(2)}\otimes T_1^{(1)}, \ C_2 = T_3^{(3)}\otimes T_1^{(1)};\\ C_3^\dagger &= T_2^{(1)}\otimes T_1^{(2)}, \quad C_3 = T_3^{(1)}\otimes T_1^{(3)};\\ C_4^\dagger &= \omega T_2^{(1)}\otimes T_2^{(2)}, \ C_4 = T_3^{(1)}\otimes T_3^{(3)}.\end{aligned}$$

Then the Hamiltonian reads

$$\hat{H}_{123} = \alpha\left(\omega^2 T_2^{(1)}\otimes T_1^{(1)}+\omega T_3^{(1)}\otimes T_1^{(1)}\right)+\beta\left(\omega^2 T_3^{(2)}\otimes T_1^{(3)}+\omega^2 T_2^{(3)}\otimes T_1^{(2)}\right)$$
$$+\gamma\left(\omega^2 T_1^{(2)}\otimes T_1^{(3)}+\omega T_1^{(3)}\otimes T_1^{(2)}\right)+\kappa\left(\omega T_3^{(3)}\otimes T_1^{(2)}+T_2^{(2)}\otimes T_1^{(3)}\right).$$

Based on the the algebraic relation in equation (1), we try to find the independent symmetry operators without linear composition. The general form of the operators can be expressed as

$$\Gamma_{\mathrm{i}} = T_a^{(x)}\otimes T_b^{(y)}. \tag{22}$$

Here a, b, x and y are to be determined, $\{a,b,x,y\}\in\{1,2,3\}$. The condition is that each term in $\hat{H}_{123}$ commutes with Γ_{i},

$$[T_2^{(1)}\otimes T_1^{(1)}, T_a^{(x)}\otimes T_b^{(y)}] = 0;$$
$$[T_3^{(2)}\otimes T_1^{(3)}, T_a^{(x)}\otimes T_b^{(y)}] = 0;$$
$$[T_1^{(2)}\otimes T_1^{(3)}, T_a^{(x)}\otimes T_b^{(y)}] = 0;$$
$$[T_2^{(3)}\otimes T_1^{(2)}, T_a^{(x)}\otimes T_b^{(y)}] = 0.$$

After direct calculation, one obtains

$$x = 1,\ a = b\in\{1,2,3\},\ y\in\{1,2,3\}. \tag{23}$$

Totally, there are 8 cases,

$$\Gamma_1 = T_1^{(1)}\otimes T_1^{(2)}, \Gamma_5 = T_1^{(1)}\otimes T_1^{(3)};$$
$$\Gamma_2 = T_2^{(1)}\otimes T_2^{(1)}, \Gamma_6 = T_3^{(1)}\otimes T_3^{(1)};$$
$$\Gamma_3 = T_2^{(1)}\otimes T_2^{(2)}, \Gamma_7 = T_3^{(1)}\otimes T_3^{(3)};$$
$$\Gamma_4 = T_2^{(1)}\otimes T_2^{(3)}, \Gamma_8 = T_3^{(1)}\otimes T_3^{(2)}.$$

But it is easy to check that

$$\Gamma_5 \propto [\Gamma_1]^2, \quad \Gamma_6 \propto [\Gamma_2]^2,$$
$$\Gamma_7 \propto [\Gamma_3]^2, \quad \Gamma_8 \propto [\Gamma_4]^2,$$
$$\Gamma_3 = \Gamma_2\Gamma_1,\ \Gamma_4 = \Gamma_2[\Gamma_1]^2.$$

Then only 2 independent operators are left,

$$\Gamma_1 = T_1^{(1)}\otimes T_1^{(2)}, \tag{24}$$
$$\Gamma_2 = T_2^{(1)}\otimes T_2^{(1)}. \tag{25}$$

Making inverse SU(3) J-W transformation, we can redefine that

$$P = \Gamma_2 = C_1 C_2^\dagger C_3 C_4^\dagger, \tag{26}$$
$$\Gamma = \Gamma_1 = \omega C_1^\dagger C_2 C_3^\dagger. \tag{27}$$

Here we define P as the $\mathbb{Z}_3$ ω-parity operator. Hence we find the two symmetry operators of $\hat{H}_{123}$.

---

[1] Liu, M., Bai, C. M., Ge, M. L. & Jing, N. H. Generalized Bell states and principal realization of the Yangian $Y(sl_n)$, *J. Math. Phys.* **54,** 021701 (2013).

[2] Ge, M. L., Wu, Y. S. & Xue, K. Explicit trigonometric Yang-Baxterization, *Int. J. Mod. Phys. A* **6,** 3735 (1991).

[3] Chen, J. L., Xue, K., & Ge, M. L. Braiding transformation, entanglement swapping, and Berry phase in entanglement space, *Phys. Rev. A* **76,** 042324 (2007).
